# Feasibility and Acceptability Study of a Culturally Adapted Web-Based Intervention to Reduce Suicidal Ideation for Syrian Asylum Seekers and Refugees in the United Kingdom: Protocol for a Mixed Methods Study

**DOI:** 10.2196/56957

**Published:** 2024-09-02

**Authors:** Oliver Beuthin, Sadiya Shahid, Ly-Mee Yu, Kamaldeep Bhui

**Affiliations:** 1 Department of Psychiatry University of Oxford Oxford United Kingdom; 2 Department of Medieval and Modern Languages and Linguistics University of Cambridge Cambridge United Kingdom; 3 Nuffield Department of Primary Care Health Sciences University of Oxford Oxford United Kingdom; 4 Department of Pyshciatry University of Oxford Oxford United Kingdom

**Keywords:** cultural adaptation, digital mental health, suicidal ideation, refugee mental health, Syrian refugee, experience-based co-design, mental health, suicide, suicidal, refugee, immigrant, ethnic minority, asylum, user experience, cultural, Syria, Syrian, refugees, feasibility, acceptability, depression, anxiety, posttraumatic stress disorder, United Kingdom, Arabic-speaking

## Abstract

**Background:**

The war in Syria has displaced over 6.8 million people, more than any other conflict since the Second World War. As a result, Syrian asylum seekers and refugees have experienced several life-changing events, resulting in high rates of anxiety, depression, posttraumatic stress disorder, and suicidal ideation (SI). To address the treatment gap and reduce the burden of help-seeking, a web-based intervention to reduce SI developed for general populations was culturally adapted for and with Syrian asylum seekers and refugees in the United Kingdom. The study revealed the importance of understanding their lived experience with migration and the acculturative process in providing treatment for SI. This study will now assess the feasibility and acceptability of the culturally adapted intervention for this population.

**Objective:**

The first phase of the study will include recruiting participants and delivering the web-based intervention (1) to assess the feasibility of meeting recruitment goals and recruitment rates and (2) to assess the feasibility of outcome measures. The second phase of the study will include one-to-one semistructured interviews (1) to assess the suitability of the culturally adapted intervention in terms of recruitment and adherence rates and barriers and facilitators to engagement and (2) to assess the acceptability of the intervention in terms of its cultural relevance and appropriateness.

**Methods:**

This is a protocol for a single-group, noncontrolled, mixed methods feasibility and acceptability study of a culturally adapted web-based intervention to reduce SI for Syrian asylum seekers and refugees in the United Kingdom. The study will assess the feasibility of recruitment goals, recruitment rates, adherence rates, and outcome measures using individual participant tracking forms, which will be analyzed quantitatively. The suitability and acceptability of the intervention will be assessed using one-to-one semistructured interviews with 12 participants who completed the intervention, which will be analyzed qualitatively.

**Results:**

Recruitment began in February 2024 and will run until 30 participants are recruited to the study or until the end of July 2024. Thus far, 19 participants have provided informed consent, 16 were eligible and enrolled, and 12 have completed a postintervention interview. No data have been analyzed. The study, including the write-up period, is expected to end in December 2024.

**Conclusions:**

Despite experiencing several stressors related to forced displacement and high rates of mental health issues, access to treatment is still limited for Syrian asylum seekers and refugees in the United Kingdom. To address the treatment gap and reduce the burden of help-seeking, a web-based intervention to reduce SI was culturally adapted in collaboration with Syrian asylum seekers and refugees in the United Kingdom. This study will now assess the feasibility and acceptability of the intervention and culturally appropriate recruitment strategies.

**Trial Registration:**

ISRCTN ISRCTN11417025; https://www.isrctn.com/ISRCTN11417025

**International Registered Report Identifier (IRRID):**

PRR1-10.2196/56957

## Introduction

### Background and Rationale

The Syrian conflict has sparked the largest forced displacement crisis since World War II [1]. Experiences such as bombings, shootings, torture, and involuntary conscription have forced many Syrians to leave behind their homes, jobs, businesses, and extended families to find safety in other cities or abroad [2]. While most of the 6.8 million Syrian refugees living abroad found refuge in neighboring countries, over 1 million have sought asylum in Europe [3]. In the United Kingdom, over 20,000 Syrians were resettled under the Vulnerable Persons Resettlement Scheme [4]. Many more Syrians have sought asylum in the United Kingdom, including 75,615 applications during the year ending in September 2021 [3]. Despite having escaped war, Syrian asylum seekers continue to experience stressors while living in the United Kingdom including, most significantly, a prolonged asylum process [2]. These stressors have resulted in elevated rates of mental health issues including major depressive disorder, posttraumatic stress disorder (PTSD), and suicidality [5]. There is thus an urgent need to improve the understanding and care of mental health issues for Syrian asylum seekers and refugees arriving in the United Kingdom.

### Suicidal Ideation

One of the most concerning and least addressed mental health issues for asylum seekers and refugees across Europe is suicidal ideation (SI) and suicidal behavior more broadly [6]. Several studies have shown high rates of SI among asylum seekers and refugees in Sweden, 33.9% [7] and 48% [8]; Switzerland, 42.1% [9] and >30% [10]; and the United Kingdom, 40.9% [11]. Some studies, however, have shown lower rates of suicide attempts among refugees than the Swedish-born population, suggesting variances between asylum seekers and refugees [12]. In fact, a study in Switzerland found that the most frequent reason for referral to emergency psychiatric services among asylum seekers was SI after the rejection of their asylum application [9]. Studies on Bhutanese refugees in the United States have also showed lower rates of SI at 3% [13], 6.2% [14], and 6.7% [15]. However, Meyerhoff and Rohan [15] found a large disparity between their reports of SI, 6.7%, and desire for death, 48.3%, suggesting that culturally inflexible models could mask the real prevalence of SI for different asylum seeker and refugee subgroups. Most importantly, despite their risk for developing mental health issues and SI, refugees and asylum seekers continue to underuse mental health services [16-19].

### Cultural Adaptation

Access to treatment for SI among Syrian asylum seekers and refugees in the United Kingdom can be improved with culturally adapted digital mental health interventions [20]. In response to the treatment gap, Beuthin et al [21] culturally adapted a digital intervention to reduce SI with and for Syrian asylum seekers and refugees in the United Kingdom. The study built on Eylem and colleagues’ [22] cultural adaptation of van Spijker and colleagues’ [23] Leven onder Controle (literally “Living under Control”) intervention, a guided 6-week cognitive behavioral therapy–based web-based intervention to reduce SI. The intervention includes 6 modules: thinking about suicide, dealing with thoughts and feelings, thinking about the future, thinking about the self, thinking about others, and repetition and relapse. Several randomized controlled trials (RCTs) have found the intervention to be effective in reducing SI compared to treatment as usual—an information website—for general Dutch (*d*=0.2) [24] and Belgian populations (*d*=0.34) [25] but not for Australian populations [26]. The initial cultural adaptation of the intervention for Turkish immigrants in the United Kingdom and the Netherlands was found to be acceptable; however, the study was unable to recruit enough participants for a full RCT [22].

Eylem et al [22] culturally adapted the intervention in line with Bernal and colleagues’ [27] framework, which included adapting for cultural metaphors, treatment concepts and goals and the context and methods of the intervention. This framework falls within the school of thought which, based on the universality of emotional expression, asserts that the distinct signs and symptoms of psychopathology are universal. It therefore aims to maintain the integrity of the original intervention as much as possible. However, cultural adaptation based on universal signs and symptoms undermines the more fundamental role of intentions and conventions in meaning making [28]. Conversely, another framework developed by Horrell [29] aims to develop new interventions for different cultural groups. While it is certainly more situated, the framework does not consider the role of acculturation and cultural change. Beuthin et al [21] therefore synthesized these approaches by focusing on shared lived experiences that give rise to (1) core affect, including hedonic valence and physical arousal; and (2) conceptual knowledge [30]. In other words, feeling is perceiving, and perceiving is believing and vice versa [31]. This framework therefore enables researchers to identify resilient core beliefs, which in turn are important in understanding and avoiding identity crises [32] and suicidal behavior [33]. With this paradigm in mind, the cultural adaptation process was simplified using experience-based co-design, an action research method for collaboratively improving health care services and interventions [34]. This entailed interviews to understand Syrian asylum seekers’ and refugees’ cultural conceptualizations, coping-strategies, and help-seeking behavior for mental health and SI in relation to touchpoints, crucial moments that made a difference in their experience, of the migratory process [2]. After a preliminary cultural adaptation of the intervention, Syrian asylum seekers and refugees participated in co-design events to collectively identify and adapt touchpoints of the intervention. A community research panel also helped to maintain the integrity of the intervention and the users’ core beliefs at each phase of the cultural adaptation. Importantly, intentions and conventions are not understood as a spectrum, but rather, all communication by definition is intentional but differs in the level to which it adheres to conventions.

Based on the findings of Beuthin et al [2], the main cultural adaptations to the intervention included case examples, coping strategies, and a nondirective approach. Case examples included a wish for death during the post-migration phase, and SI crises during the pre- and peri-migration phases. Notably, a participant who experienced an SI crisis described his thoughts as both SI and a wish for death, potentially highlighting a more salient mechanism of SI for this population. However, while attributing their thoughts to their experiences, they believed that suicide is fundamentally a consequence of diminished sources of support including weak imān (faith), a loss of one’s mind, character and soul, and a lack of access to mental health support. Similarly, participants identified their coping strategies as conceptualizing their experiences as ibtilā’ (a test) and being hopeful of God’s help while engaging in acts of worship, hobbies, walking, volunteering, talking to family and friends, etc. In contrast to the previous feasibility study by Eylem et al [22] that suggested the intervention should be shortened, participants preferred maintaining the integrity of the intervention while providing more explanations about their purpose [2]. Participants were also directly involved in the cultural adaptation process through 3 co-design events, which involved adapting 2 sessions of the intervention each. However, since participants did not use the intervention, this study will now assess its acceptability and suitability by providing it in full to Syrian asylum seekers and refugees in the United Kingdom with SI.

### Recruitment

In addition to the cultural adaptation of the intervention, Eylem et al [22] expressed the need to identify culturally appropriate recruitment strategies. Despite advertising their study through public events, newspaper advertisements, television programs, banners on websites, and social media, they were unable to recruit enough participants for a full RCT. They believed that recruitment was hindered by institutional barriers for different Turkish subgroups and therefore recommended task-shifting recruitment to trained lay people from the target population. This was confirmed in a previous study by Beuthin et al [2], which recruited all its participants through cultural brokers via the web. Most charities and nongovernmental organizations (NGOs) contacted in the previous study reported having insufficient capacity to disseminate the study advert. Likewise, participants of the study reported avoiding seeking help via the National Health Service (NHS) due to the long waiting times and fear of having their asylum application rejected. Several Syrian mental health researchers in the United Kingdom confirmed that despite holding events and advertising through mosques, charities, and NGOs, they too only received responses via advertisements in Syrian diaspora Facebook groups. Thus, this study will assess the feasibility of recruiting Syrian asylum seekers and refugees in the United Kingdom with SI from various sources including charities, NGOs, local councils, social media, but most importantly, cultural brokers.

### Aims

This study is part of a broader research project aimed at improving the understanding and treatment of SI among Syrian asylum seekers and refugees arriving in the United Kingdom. The study aims to investigate the feasibility and acceptability of a culturally adapted web-based stand-alone intervention to reduce SI for Syrian asylum seekers and refugees in the United Kingdom.

### Objectives

The first phase of the study will include recruiting participants and delivering the digital intervention (1) to assess the feasibility of meeting recruitment goals and recruitment rates and (2) to assess the feasibility of outcome measures. The second phase of the study will include one-to-one semistructured interviews (1) to assess the suitability of the culturally adapted intervention in terms of recruitment and adherence rates as well as barriers and facilitators to engagement and (2) to assess the acceptability of the culturally adapted intervention in terms of its cultural relevance and appropriateness and whether participants were satisfied with their experience.

## Methods

### Study Design

This is a single-group, noncontrolled, mixed methods feasibility and acceptability study of a culturally adapted web-based intervention to reduce SI for Syrian asylum seekers and refugees in the United Kingdom. The feasibility and acceptability study will follow the SPIRIT (Standard Protocol Items: Recommendations for Interventional Trials) 2013 Guidance for protocols of clinical trials [35] and the CONSORT (Consolidated Standards of Reporting Trials) 2010 extension for randomized pilot and feasibility trials [36].

Expanding on the feasibility study by Eylem et al [22] of the same intervention, the study will assess the feasibility of recruitment methods in meeting recruitment goals. Previously, Eylem et al [22] aimed to conduct a full RCT; however, they were unable to meet their recruitment goal of 286 participants, recruiting instead 85 participants, 30 of whom were found to be eligible, and only 18 provided consent. They therefore recommended task-shifting to trained lay people or cultural brokers to overcome institutional barriers to recruitment.

The feasibility of recruitment goals and recruitment rates will be assessed in accordance with the guidelines for evaluating the feasibility of recruitment in pilot studies of diverse populations developed by Stewart et al [37]. Their guidelines include the following eight steps: (1) specifying recruitment goals (target population, desired diversity, and subgroup sample sizes), (2) specifying recruitment processes by stage, (3) establishing a tracking system for each individual (contact tracking form), (4) establishing a tracking database for monitoring processes and results, (5) implementing recruitment processes and monitoring each individual’s progress, (6) summarizing recruitment results from the tracking database, (7) calculating and interpreting measures of feasibility, and (8) modifying methods for a larger study if recruitment goals are not met. Their guidelines will be described in more detail in the Data Collection, Management, and Analysis section.

Like the feasibility study by Eylem et al [22], one-to-one semistructured interviews will be conducted with intervention participants to understand the suitability and acceptability of the culturally adapted intervention. This will include identifying the barriers and facilitators to engagement and the cultural appropriateness and relevance of the culturally adapted intervention. Like the initial cultural adaptation process for Syrian asylum seekers and refugees in the United Kingdom, the interviews will involve asking participants to identify touchpoints of the intervention [34]. Research methods will be described in more detail in the Data Collection, Management, and Analysis section.

### Participants, Interventions, and Outcomes

#### Setting

As Syrian asylum seekers and refugees are dispersed across the United Kingdom, all study activities including recruitment, consent, screening for eligibility, intervention, and interviews will be held on the web to lower barriers to participation. However, access to the intervention could still be influenced by barriers and facilitators associated with its institutional affiliations including the University of Oxford, which will be mentioned on all foreword-facing documents, and those who agree to advertise it to their network.

#### Eligibility Criteria

Inclusion criteria include being a male or female Syrian asylum seeker or refugee in the United Kingdom, aged 18 years and older, and with at least a score of 1 on the Columbia-Suicide Severity Rating Scale (C-SSRS) [38]. Participants also need to be internet literate, have access to a PC or mobile phone with internet, and be willing to provide their email address and the email address of their general practitioner (GP) surgery. Recruitment will take place via the web; and therefore, internet literacy and access also apply. Participants with a score of 4 or above on the C-SSRS, indicating serious SI, will not be eligible to participate. This decision was made to avoid risks of harm to the participants and has thus far not resulted in the exclusion of any participants.

#### Interventions: Description

After providing informed consent and screening for eligibility, baseline measurements of SI, anxiety, depression, and PTSD will be taken for intervention participants. Participants will then receive the 6-week self-help digital intervention via Qualtrics (Qualtrics International Inc), which consists of the following 6 sessions [22]:

1. Thinking about suicide: The aim of this module is to enable participants to identify the automatic thoughts that give rise to suicidal thoughts in order to gain some control over their thoughts. Specific attention is given to the dichotomous and overgeneralizing nature of the thoughts.
2. Dealing with thoughts and feelings: Participants will learn that they can be resilient in the face of seemingly unbearable feelings. Focus will be given to learning how to tolerate and regulate intense emotions in crisis situations.
3. Thinking about the future: Participants will evaluate their ideas about the future and ask whether they are realistic. Consequently, participants will develop a more realistic view of the future and set new goals.
4. Thinking about the self: Common mistakes in thinking about the self are discussed and challenged. Participants are also taught how to manage suicidality as a long-term vulnerability. Particular attention is given to learning to seek help.
5. Thinking about others: Mistakes in thinking about others will be discussed. Participants will also be provided with information about the effects of suicide for relatives and friends.
6. Repetition and relapse: Several mistakes in thinking and acquired skills are repeated. Attention will also be given to relapse and how to avoid it.

While the intervention was initially intended as a 6-week intervention, it will be provided to participants as a 6-session intervention, which they can complete at their own pace within 6 weeks. Preferably, this involves working on the intervention for 15 minutes daily. If participants fail to submit the session’s homework by the end of each week, a further message will be sent asking if they are having any difficulties or if they want to discontinue their participation. If the participant does not reply within a week, they will be contacted again when it is time to assess their SI as part of the study’s safety protocol. If they do not respond, it will be assumed that they no longer wish to participate. Likewise, any participant who scores 4 or above on the C-SSRS will be excluded from participation [38]. All participants will be encouraged to seek help from their GP or will be provided information about alternative sources of help during the intervention.

While the goal of the study is to track rather than ensure adherence, strategies for improving adherence were one of the main objectives of the cultural adaptation. Specifically, the electronic case tracking form (eCTF) used to track recruitment goals will be used to track the reasons for ending participation throughout the intervention. Adherence rates, in terms of the number of sessions completed and the amount of time taken to complete them, will be assessed during postintervention interviews and with the platform user analytics.

#### Outcomes

##### Primary Outcomes

The primary outcomes of the study include the acceptability and suitability of the intervention to Syrian asylum seekers and refugees in the United Kingdom with SI. The acceptability of the intervention to participants will be assessed during interviews in terms of cultural relevance (ie, the familiarity of the therapeutic content to one’s cultural background and lived experiences with the migratory process), cultural appropriateness (ie, appropriateness of the therapeutic content in terms of the Syrian cultural context), and whether participants were satisfied with their experience [22].

The suitability of the intervention will be analyzed quantitatively in terms of recruitment and adherence rates. Recruitment rates will be assessed in terms of the percentage of those contacted who met the eligibility criteria and the percentage of those eligible who were enrolled, which should be 70% at each stage. Adherence rates will be assessed in terms of the number of sessions attended, which should be at least 70%, and the time spent completing them. The suitability of the intervention will also be assessed qualitatively in terms of the barriers and facilitators of engagements by way of the case tracking form and postintervention interviews.

##### Secondary Outcomes

Secondary outcomes include the feasibility of recruitment goals and measurement instruments. Recruitment goals will be tracked in accordance with the guidelines by Stewart et al [37] for diverse populations. This will include using an eCTF to track the sources of potential participants (NGOs, charities, and social media), the number of participants recruited within the set time period (30 in 6 months), sample characteristics (age, gender, ethnicity, and religion), and reasons for loss at each stage (consent, eligibility, enrollment, etc).

The feasibility of measurement instruments will be assessed in terms of the number of people who completed questionnaires, the amount of information missing, which should not exceed 10%, and the time it took to complete them. This will include measuring SI for intervention participants at baseline (T1), after 3 weeks (T2), and post treatment (6 weeks after baseline: T3). Depression, anxiety, and PTSD will also be measured at baseline and post treatment. If possible, all participants who begin the intervention will complete the follow-up assessment at week 6 irrespective of whether they complete the intervention or not. All the data will be recorded on the eCTF. The outcome measures used in the study are as follows:

SI will be measured using the SI subscale of the C-SSRS, which is a 6-item tool used to assess SI [38], which has shown good internal consistency (α=.966) for an Arabic-speaking population [39]. The C-SSRS was chosen due to its compatibility with Syrian’s conceptualization of SI as a wish for death. An answer of yes to any of the 6 questions indicates a presence of SI, and a score of 4 and above indicates serious SI.

Anxiety and depression will be measured with the Hospital Anxiety and Depression Scale (HADS) [40,41]. The Arabic version has shown acceptable internal consistency for both the anxiety (α=.73) and depression scale (α=.77) [42]. HADS consists of 14 items, which are scored on a 4-point Likert scale between 0 and 3. The total score range is between 0 and 21, with scores between 0 to 7 indicating normal anxiety or depression, scores between 8 to 10 indicating borderline abnormal anxiety or depression, and scores above 11 indicating abnormal anxiety or depression.

PTSD will be measured with the Arab versions of the Posttraumatic Stress Disorder Checklist for *DSM-5* (*Diagnostic and Statistical Manual of Mental Disorders* [Fifth Edition]; PCL-5), which has shown high (α=.85) internal consistency [43]. The scale consists of 20 self-report items. Each item includes 5 points with scores ranging between 0=not at all and 4=extreme. A total score of 31-35 or higher suggests that a patient may benefit from PTSD treatment.

### Participant Timeline

Recruitment will be conducted over a 6-month period starting from February 2024 and ending in July 2024. Recruitment goals will be recorded using the case tracking form up until a final disposition is made. As soon as participants contact OB, they will receive a participant information sheet (PIS) and schedule a time to provide consent. The informed consent process will take 10 minutes via Microsoft Teams, during which the participant will be able to ask questions, and written informed consent can be completed on their behalf. An additional 5 minutes is needed to screen the participant for eligibility. Eligible participants will then complete baseline measures for SI, anxiety, depression, and PTSD, which should take 15 minutes. This entire process should take no longer than 30 minutes. As part of the study’s safety protocol, participants will be contacted at week 3 and 6 to assess their level of SI, which should only take 5 minutes. After 6 weeks, a prearranged web-based meeting will be held via Microsoft Teams to take postintervention measures of SI, anxiety, depression, and PTSD, which should require 15 minutes to complete. Intervention participants will also be sent an email to schedule a date for an interview within 1 week of completing the intervention. The interview will be held via Microsoft Teams, which should take 45-60 minutes to complete.

### Sample Size

The study aims to recruit 30 male or female Syrian asylum seekers and refugees in the United Kingdom, aged 18 years and older, with at least a score of 1 on the C-SSRS [38]. To assess the acceptability of the intervention, at least 12 eligible participants must be recruited for the intervention and postintervention interviews within 6 months (February to July 2024). This number was chosen to enable data saturation for the acceptability and suitability interview at 7 participants while compensating for expected attrition. Since the target population are minorities in the United Kingdom, recruitment is expected to have a lower yield within the allocated time period. If possible, participants will be stratified for sex (male: n=6 and female: n=6), major ethnic groups (Arab: n=10 and Kurdish: n=2), and age (18-39 years: n=10 and 40 years and older: n=2). The National Center for Complementary and Integrative Health notes that sample sizes should be based on practical considerations, including participant flow, financial constraints, and the number of participants needed to evaluate feasibility goals [44]. For qualitative work, 30 or less participants are enough to reach saturation.

### Recruitment

As mentioned before, Eylem et al [22] were unable to recruit enough participants for an RCT and therefore recommended task-shifting to lay people associated with the target population. Task-shifting recruitment to community-based health workers and volunteers has shown to improve recruitment and access for minority ethnic groups [45,46] and was also suggested to scale up mental health interventions for Syrian refugees in the Middle East and Europe [47]. Thus, participants will be recruited via cultural brokers as well as NGOs, charities, local councils, and social media.

The study will use purposive sampling to recruit participants from various demographic backgrounds including age, gender, religion, ethnicity, and refugee status. All recruitment will be via the web using a study advertisement or email correspondence, which will outline the purpose and development of the intervention. OB will be responsible for all direct communication with participants and external individuals and organizations involved in sharing information about the study. The recruitment process will be recorded using an eCTF in accordance with the 8-step guidelines of Stewart et al [37], which includes tracking the sources of potential participants (NGOs, charities, local councils, and social media), the number of participants recruited within the set time period (60 in 6 months), sample characteristics (age, gender, ethnicity, and religion), and reasons for loss at each stage (consent, eligibility, randomization, waiting list, intervention, and interview). No incentives for participation will be provided other than access to the intervention.

### Assignment of Interventions

Since this is an open-label study, aspects such as randomization and blinding procedures were not considered.

### Data Collection, Management, and Analysis

#### Data Collection Plan

##### Primary Outcomes

In line with the feasibility study by Eylem et al [22] of the same intervention, the acceptability of the intervention to participants will be assessed using one-to-one semistructured interviews in terms of cultural relevance (ie, the familiarity of the therapeutic content to one’s cultural background), cultural appropriateness (ie, appropriateness of the therapeutic content in terms of the Syrian cultural context), and whether participants were satisfied with their experience. Like the previous study [2], participants’ lived experience with the migratory process will form the basis of discussions on cultural relevance and appropriateness.

As mentioned earlier, the suitability of the intervention will be analyzed quantitatively in terms of recruitment and adherence rates. Data will be collected with case tracking forms throughout the intervention and during interviews at the end of the intervention. Self-reported and user analytics adherence rates will assess the acceptability of these adaptations and the need for further adaptations.

##### Secondary Outcomes

Recruitment goals will be tracked in accordance with the guidelines of Stewart et al [37] for diverse populations. The use of their guidelines will not only provide structure to the recruitment process but will enable its replication for future studies.

The feasibility of measurement instruments will involve measuring SI for intervention participants at baseline (T1), after 3 weeks (T2), and post treatment (6 weeks after baseline: T3) and depression, anxiety, and PTSD at T1 and T3. Measurements will all be completed during web-based meetings and recorded on the eCTF.

In total, participants will be assessed using 3 outcome measures: C-SSRS [38], the Arabic version of the HADS [40,41], and the Arabic version of the PCL-5 [43]. The C-SSRS was chosen due to its assessment of both SI and a wish for death as well as its good internal consistency (α= .966) for an Arabic-speaking population [39]. The HADS [40,41] was chosen due to its Arabic version showing acceptable internal consistency for both the anxiety (α=.73) and depression scale (α=.77) [42]. In a previous study, Beuthin et al [2] identified stressors with the asylum process as a major concern for Syrian asylum seekers in the United Kingdom, which could be related to anxiety and depression. Finally, the Arabic version of the PCL-5 has shown high (α=.85) internal consistency [43]. Again, in the study by Beuthin et al [2], several Syrian refugees in the United Kingdom reported being diagnosed with PTSD.

##### Quality Control

All participant data, except for interviews, will be recorded in a separate eCTF, which will be completed in an Excel sheet kept in OneDrive for Business. Data from eCTFs will be added to a joint database for analysis once the participant reaches a final outcome. Interviews will be audio-recorded to ensure accuracy. Once transcribed and translated, the audio recordings will be deleted. At the end of the study, all data will be transferred to the chief investigator.

##### Training

All members of the research team have completed the National Institute for Health and Care Research Good Clinical Practice course as well as internal university training in research integrity and security and data privacy awareness. All intervention coaches will be fluent Arabic speakers with at least a master degree in health sciences. They will be trained by the DPhil researcher (OB) in the purpose and procedures of each intervention component to help answer participant questions and provide feedback on their homework.

#### Data Collection Plan: Retention

##### Participant Retention

To enable retention, obtaining informed consent, eligibility screening, enrollment, and all outcome measures will be completed via Microsoft Teams with the assistance of OB instead of web-based surveys. Participants will be followed throughout the trial, and reasons for nonadherence and withdrawal will be recorded within the eCTF if possible. As mentioned earlier, the intervention has been culturally adapted to improve engagement and understanding [2].

##### Participant Withdrawal

Participants will be informed in the PIS that if they choose to discontinue their participation, information recorded in the eCTF up until discontinuation will be retained. All other data including their contact details and those of their GP surgery, exercises and interview audio recording, and transcriptions and translations will be deleted. Participants who score above the cutoff score (4 and above) on the C-SSRS [39] indicating active SI with some intent to act at T2 (week 3), and T3 (week 6) will be withdrawn. To ensure participation in postintervention interviews, the time and date for the interview will be set with the participant during T2 measurements of SI.

##### Data Management

Original written informed consent forms and outcome measures completed by OB on behalf of the participant will be scanned, and password-protected copies will be sent to the participant via email. These data as well as eligibility screening and data necessary to stratify the postintervention interviews such as ethnicity, gender, and religion will be entered electronically into individual case tracking forms labeled with a unique participant number and held in OneDrive for Business provided by the university. Data from eCTFs will later be added to a joint database for analysis once the participant reaches a final outcome. Interview participants’ audio recordings will be labeled with a unique participant number and stored on OneDrive for Business. They will then be deleted after their transcriptions and translations are uploaded to OneDrive. Participant contact details connected to their unique participant number will be kept in a form on OneDrive for Business throughout their participation and deleted when they reach a final outcome. Access to the uploaded data will only be permitted for the DPhil researcher (OB) and the principal investigator (KB) and upon request to members of the Medical Sciences Interdivisional Research Ethics Committee for the purposes of monitoring and auditing of the research. In accordance with university policy, all nonidentifiable data will be stored for at least 3 years after publication. At the end of the study, OB will transfer all data from university servers to the custodianship of the principal investigator (KB), who will thereon be responsible for data management.

##### Quantitative Outcomes

Quantitative feasibility outcomes will be reported descriptively. Recruitment rates will be assessed in terms of the percentage of those contacted who met the eligibility criteria and the percentage of those eligible who were enrolled. Adherence is defined in terms of the number of sessions attended and the time spent completing them. Adherence rates will be calculated as the percentage of all participants who adhered to the intervention. Outcome measure completion rates are defined as the percentage of participants who completed the outcomes and the percentage of the outcomes completed. The feasibility of recruitment methods meeting recruitment goals will also be assessed quantitatively in terms of the percentage of participants recruited from different sources (NGOs, charities, social media, and cultural brokers), the number of participants recruited within the set time period, and the sample characteristics (age, gender, ethnicity, and religion).

##### Qualitative Outcomes

The suitability of the intervention will also be assessed qualitatively in terms of the barriers and facilitators to engagement. This will include participants’ reasons for attrition recorded in the eCTF and key touchpoints of their experience with the intervention. Acceptability of the intervention in terms of cultural relevance and appropriateness and participant’s overall satisfaction with the intervention will be analyzed thematically. Building on the previous study to culturally adapt the digital intervention, the thematic analysis will be conducted in accordance with the 6 phases outlined by Braun and Clarke [48]. Importantly, Braun and Clarke [48] differentiate between semantic themes revealed through description and latent themes through interpretation. This understanding of language is based on a distinction between semantics and pragmatics, which limits meaning to adherence with conventions. However, since language is a social practice, meaning is more fundamentally the use of conventions [28]. This understanding is especially clear where conventions differ between interlocutors such as in intercultural communication. Therefore, this study will instead extract themes based on the communication of touchpoints of lived experience and the objectives of the study [28]. Internal reliability will be ensured by an iterative review of themes as well as checking for consistency between members of the research team [49]. Irreconcilable differences in interpretations of the data will be solved by preferencing interpretations in line with participants’ experiences with the intervention and the objectives of the interviews.

### Monitoring

#### Harms

The intervention was previously found to be safe for general populations in the Netherlands [24], Belgium [25], and Australia [26] and Turkish immigrants in the Netherlands and the United Kingdom [22]. A safety protocol developed in collaboration with and approved by the university ethics department will ensure the safety of participants throughout the study. The safety protocol has an important balance to make between minimizing the risk of adverse events while still ensuring that the intervention is provided to populations that are most likely to use it. As such, Syrian asylum seekers and refugees in the United Kingdom who have serious SI will not be allowed to participate. After providing consent, participants will be assessed for suicidal thoughts using the SI subscale of the C-SSRS [38]. An answer of yes to any of the 6 questions indicates a presence of SI and a score of 4 and above indicates serious SI, specifically, active SI with some intent to act. Participants who answer yes to any of the questions will be encouraged to contact their GP and will be provided with information about alternative sources of help. In addition to measuring SI before receiving the intervention (T1: baseline), SI will be measured again at T2 (week 3) and T3 (week 6). Any increase in SI will be reported to the principal researcher (KB), who will review the circumstances and act according to his judgment. If a participant scores above the cutoff score (4 and above) during the web-based meeting, they will be immediately encouraged to seek help from their GP or their GP surgery will be contacted with a generic email already approved by the ethics committee, and they will be withdrawn from the study. Participants will be informed about this procedure in the PIS.

The DPhil researcher (OB) will also report any incidental findings to the principal investigator (KB), who will in turn review the circumstances and act according to his judgment. If further steps are necessary, the matter will be referred to the Multi-Agency Safeguarding Hub, which will be made within 1 working day of first receiving the information from the participant. The Local Area Designated Officer will then advise on the next appropriate steps to take. If any risk of harm is directed toward an adult, a referral will be made to the relevant statutory agency. If there is a risk of immediate harm, the emergency services will be contacted via 999.

#### Auditing

The Medical Sciences Interdivisional Research Ethics Committee at the University of Oxford will have access to the study data upon request for the purposes of monitoring and auditing of the research.

### Ethical Considerations

Ethics approval has been provided for the study by the Central University Research Ethics Committee at the University of Oxford (R89074/RE001). Eligible participants will receive a PIS before meeting via Microsoft Teams to ask questions and provide their informed consent. The PIS will describe, in both Arabic and English, the goals and limitations of the study, its procedures including the intervention and how data will be collected and handled, and any risks to participants. Participants will also be told that they are free to withdraw from participation at any time, for any reason, and without consequence. Since the intervention is web-based, written informed consent will be completed on behalf of the participant. A digital copy will be provided to the participant and kept on university servers for at least 3 years from the time of publication. Access to personal data will be restricted to only authorized individuals. Personal names will be replaced with unique participant numbers on eCTFs and audio recordings and transcriptions of interviews. All audio recordings will be deleted once they have been transcribed, and all original paper copies will be destroyed once transferred to eCTFs. The remaining data will be kept on university servers, OneDrive for Business, for 3 years from the time of first publication in accordance with Oxford University policy. Like the previous study [2], the findings of this study will be disseminated through publication and web-based Syrian asylum seeker and refugee communities. The overall research project will contribute toward OB’s dissertation research, which will be held in Oxford University’s Bodleian Library and possibly published at a later stage. As per the stipulations of the ethics committee, participants will not be provided any compensation for their involvement in the study.

## Results

Recruitment began in February 2024 and will run until 30 participants have been recruited to the study or until the end of July 2024. Thus far, 19 participants have been recruited and provided consent, 16 of whom were eligible, and 12 have completed the postintervention interview. All but one participant were recruited via cultural brokers. The study, including the write-up period, is expected to end in December 2024.

## Discussion

### Expected Findings

This paper describes the study protocol for the feasibility and acceptability study of a culturally adapted digital SI intervention for Syrian asylum seekers and refugees in the United Kingdom. The intervention was previously culturally adapted in collaboration with Syrian asylum seekers and refugees in the United Kingdom. First, the adaptation process involved conducting interviews to explore their lived experiences with the migratory process; their cultural conceptualizations, coping strategies, and help-seeking behavior for mental health and SI; and their attitudes toward digital mental health. One of the most significant findings of this phase was that a wish for death may not only be less stigmatized but is also a more salient mechanism of SI for this population. More specifically, a wish for death could be related to the acquired capability for suicide dimension in the interpersonal theory of suicide [50]. Moreover, while attributing their thoughts to lived experience, participants believed suicide was fundamentally caused by diminished sources of support such as weak imān (faith), a loss of one’s mind, character and soul, and a lack of mental health support. This could suggest that suicide is attributable to a lack of access to one’s repetitive sources of support. Second, Syrian asylum seekers and refugees in the United Kingdom collaborated in co-design events to identify and culturally adapt touchpoints of the intervention. The main adaptations to the intervention included translating the intervention to Arabic and adding cultural idioms of distress, case examples of a wish for death and an SI crisis, culturally relevant coping strategies, a nondirective approach that included explanations for each exercise, encouragement, and reminders throughout the intervention. For a more detailed description of the cultural adaptations, see Beuthin et al [2].

Since the participants involved in the cultural adaptation process did not use the intervention and some did not have SI, further research is needed to assess its feasibility and acceptability to users with SI. Thus, this study will consist of two phases: (1) recruiting participants and delivering the digital intervention and (2) one-to-one semistructured interviews. First, participants will be recruited from various sources including cultural brokers via the web, NGOs, and charities to assess the feasibility of meeting recruitment goals and recruitment rates. Second, interviews will be conducted to assess the suitability and acceptability of the culturally adapted intervention. Findings from the study could result in further adaptations to the intervention including those already made as well as inform the design of a future RCT. Two areas that are most likely to require further adaptation are the Arabic translation and content on SI. As English and Arabic belong to very distant language families, it can be challenging to translate in either direction. Likewise, the extent to which Syrian asylum seekers and refugees are willing to approach their thoughts about suicide directly is still unknown. The findings of the study may also indicate the need to reduce the number of outcome measures or questions to be asked in a future RCT as well as the amount of contact with researchers.

### Limitations

The study has 2 main limitations. First, recruitment strategies will not include recruiting participants via the NHS. However, given the barriers to help-seeking for Syrian asylum seekers and refugees and the current budget of this study, the expected recruitment potential of the NHS does not justify the financial costs. Second, the study will exclude Syrian asylum seekers and refugees with serious suicidal thoughts from participation. While this might be a necessary safety precaution for this study, Syrian asylum seekers and refugees tend toward 2 extremes in relation to SI, either a wish for death or an SI crisis. Thus, in addition to recruiting via cultural brokers, a future RCT or actual implementation of the intervention could include participants with serious SI by recruiting via the NHS.

### Conclusions

War in Syria has displaced over 6.8 million people, more than any other conflict since the Second World War [1]. As a result, Syrian asylum seekers and refugees have experienced several life-changing events including the loss of family members, property, and livelihood. These experiences have also negatively impacted their mental health including high rates of anxiety, depression, and PTSD. Syrian asylum seekers and refugees continue to experience stressors in the process of seeking refuge and help for their mental health that could result in SI [2]. However, there is currently only 1 intervention that directly addresses suicidal behavior for asylum seekers and refugees. Thus, towards closing the treatment gap, this study will assess the feasibility and acceptability of a culturally adapted digital intervention to reduce SI for Syrian asylum seekers and refugees in the United Kingdom. This includes assessing the feasibility of recruitment strategies such as task-shifting to trained lay people within the British Syrian diaspora. Identifying and understanding the touchpoints of experience associated with the intervention could also facilitate a future RCT and local implementation. Finally, this study may also provide insight into the extent to which the culturally adapted intervention can be generalized to Syrian asylum seeker and refugee populations outside of the United Kingdom.

## Abbreviations

CONSORT: Consolidated Standards of Reporting Trials
C-SSRS: Columbia-Suicide Severity Rating Scale
DSM-5: Diagnostic and Statistical Manual of Mental Disorders (Fifth Edition)
eCTF: electronic case tracking form
GP: general practitioner
HADS: Hospital Anxiety and Depression Scale
NGO: nongovernmental organization
NHS: National Health Service
PCL-5: Posttraumatic Stress Disorder Checklist for DSM-5
PIS: participant information sheet
PTSD: posttraumatic stress disorder
RCT: randomized controlled trial
SI: suicidal ideation
SPIRIT: Standard Protocol Items: Recommendations for Interventional Trials

## Data Availability

The data sets generated and analyzed during this study are not publicly available but are available from the corresponding author on reasonable request.
